# Integrated omics analysis reveals the epigenetic mechanism of visceral hypersensitivity in IBS-D

**DOI:** 10.3389/fphar.2023.1062630

**Published:** 2023-03-16

**Authors:** Yaoyao Lu, Yuna Chai, Jianli Qiu, Jingmin Zhang, Menglin Wu, Zhe Fu, Yongfu Wang, Chongzhen Qin

**Affiliations:** ^1^ Department of Pharmacy, The First Affiliated Hospital of Zhengzhou University, Zhengzhou, Henan, China; ^2^ Department of Pediatrics, The First Affiliated Hospital of Henan University of Chinese Medicine, Zhengzhou, Henan, China; ^3^ Department of General Pediatric Surgery, The Third Affiliated Hospital of Zhengzhou University, Zhengzhou, Henan, China; ^4^ Department of Chinese Medicine, The First Affiliated Hospital of Zhengzhou University, Zhengzhou, Henan, China

**Keywords:** IBS-D, visceral hypersensitivity, epigenetics, miRNAs, mRNAs, proteomics

## Abstract

**Background and objective:** IBS-D is a common functional bowel disease with complex etiology and without biomarker. The pathological and physiological basis of IBS-D focuses on visceral hypersensitivity. However, its epigenetic mechanism remains elusive. Our study aimed to integrate the relationship between differentially expressed miRNAs, mRNAs and proteins in IBS-D patients in order to reveal epigenetic mechanism of visceral hypersensitivity from transcription and protein levels and provide the molecular basis for discovering biomarkers of IBS-D.

**Methods:** The intestinal biopsies from IBS-D patients and healthy volunteers were obtained for high-throughput sequencing of miRNAs and mRNAs. The differential miRNAs were selected and verified by q-PCR experiment followed by target mRNA prediction. Biological functions were respectively analyzed for target mRNAs, differential mRNAs and the previously identified differential proteins in order to explore the characteristic involved visceral hypersensitivity. At last, interaction analysis of miRNAs, mRNAs and proteins was performed for the epigenetic regulation mechanism from transcription and protein levels.

**Results:** Thirty-three miRNAs were found to be differentially expressed in IBS-D and five of them were further confirmed, including upregulated hsa-miR-641, hsa-miR-1843, hsa-let-7d-3p and downregulated hsa-miR-219a-5p, hsa-miR-19b-1-5p. In addition, 3,812 differential mRNAs were identified. Thirty intersecting molecules were found from the analysis on the target mRNAs of miRNAs and mRNAs. Fourteen intersecting molecules were obtained from the analysis on the target mRNAs and proteins, and thirty-six intersecting molecules were identified from analysis on the proteins and different mRNAs. According to the integrated analysis of miRNA-mRNA-protein, we noticed two new molecules COPS2 regulated by hsa-miR-19b-1-5p and MARCKS regulated by hsa-miR-641. Meanwhile some critical signaling pathways in IBS-D were found such as MAPK, GABAergic synapse, Glutamatergic synapse, and Adherens junction.

**Conclusion:** The expressions of hsa-miR-641, hsa-miR-1843, hsa-let-7d-3p, hsa-miR-219a-5p, and hsa-miR-19b-1-5p in the intestinal tissues of IBS-D patients were significantly different. Moreover, they could regulate a variety of molecules and signaling pathways, which were involved in the multifaceted and multilevel mechanism of visceral hypersensitivity of IBS-D.

## 1 Introduction

Irritable bowel syndrome (IBS) is a chronic disease with rising annual morbidity worldwide (Lovell and Ford, 2012). Diarrheal IBS (IBS-D) is a typical subtype and triggered by many factors like diet, stress, gut barrier, microbiota, psychology, genetics and so on. The visceral hypersensitivity is the primary pathological basis of IBS-D (Study Group of Functional Gastrointestinal Disorders, 2020). It has been proved that various factors were involved in visceral hypersensitivity of IBS-D such as neuromodulation, immune activation, intestinal permeability, 5-hydroxytryptamine and intestinal flora. However, related epigenetic mechanisms have not been fully elucidated.

Omics technologies, like proteomics and transcriptomics, play a significant role in studying the epigenetic mechanisms of diseases. Some of the latest reports of epigenetic information in intestinal diseases have hinted the underlying mechanism of visceral hypersensitivity, and provided the possibility for the discovery of new biomarkers of IBS-D. Non-coding RNA (ncRNA), as an essential component of epigenetics, has been proved to significantly affect gut function (Gu et al., 2021; Mahurkar-Joshi and Chang, 2020; Martinez et al., 2017; Zhang Y et al., 2020). MicroRNAs (miRNAs) are the most widely studied ncRNAs and could impact RNA translation and degradation processes by sequence complementation (Wei X et al., 2021). Reports in recent years have shown that miRNAs could change the intestinal barrier and influence gut microbiota (McKenna et al., 2010; Rashid et al., 2020). Low expression of miR-19b in Crohn’s disease worsened the intestinal inflammatory response (Cheng et al., 2015), and miR-199a-3p oligomer improved intestinal barrier dysfunction in ulcerative colitis by inhibiting the IL-17A/IL-23 axis (Guo et al., 2021). The latest report showed that the miRNA profile of IBS-D patients differed from that of healthy volunteers (Bravo-Vazquez et al., 2022). However, further epigenetic regulation of miRNAs is needed to be confirmed in the visceral hypersensitivity occurring in IBS-D. Integrated omics analysis has significant advantages of multidimensionality and systematization in the pathogenesis exploration of diseases. Combined analysis of miRNA-mRNA-protein in IBS-D patients can better elucidate the epigenetic process of visceral hypersensitivity.

In a previous study, we fully analyzed the protein profile of IBS-D patients’ intestinal tissues by tandem mass spectrometry tagging (TMT) proteomics and found many significant molecules (Chai et al., 2021). However, the upstream miRNA/mRNA regulatory mechanisms on these proteins need to be further investigated. In this study, we identified differentially expressed miRNAs and mRNAs. Then bioinformatics functions of target mRNAs of miRNAs, differential mRNAs and proteins were revealed. What’s more, we integrated different omics data of IBS-D and got many intersecting molecules as well as important signaling pathways, which provided the molecular basis for the epigenetic mechanism of visceral hypersensitivity in IBS-D.

## 2 Results

### 2.1 Clinical characteristics of patients

Five IBS-D patients and five healthy volunteers were selected from the recruited volunteers who met the diagnostic criteria for omics study. Clinical characteristics were displayed in Table 1. There were no significant differences in age, sex, weight, height, or BMI between the IBS-D group and control group (*p* > 0.05). The scores of clinical symptoms showed overall severity of abdominal pain, abdominal distension and diarrhea were moderate. None of the subjects took any medication.

**TABLE 1:** The clinical data of subjects.

	IBS-D (*n* = 5)	Control (*n* = 5)	*p*-value
Age (year)	32.2 ± 12.4	36.2 ± 9.5	0.50
BMI (kg/m^2^)	21.8 ± 3.7	21.7 ± 1.9	0.15
Weight (kg)	64.0 ± 10.9	58.2 ± 8.3	0.25
Height (cm)	171.2 ± 6.9	163.4 ± 7.1	0.88
Female/male	1/4	3/2	—
Duration (year)	5.0 ± 5.5	—	—
Defecating frequency^a^	1.4 ± 0.5	—	—
Stool consistency^b^	2.4 ± 0.5	—	—
Abdominal distension^c^	0.8 ± 0.8	—	—
Intensity of abdominal pain^d^	1.0 ± 0	—	—
Frequency of abdominal pain^e^	1.8 ± 1.3	—	—

*They are represented by scores based on the detailed scoring criteria:

^a^ Defecating frequency is scored based on the average number of bowel movements per day in the last week.

^b^ Stool consistency is scored based on the Bristol stool pattern score (0 score for type 4, 1 score for type 5, 2 score for type 6 and 3 score for type 7).

^c^ Abdominal distension is scored on a scale of 0–4 according to severity.

^d^ Intensity of abdominal pain score according to visual analog scale (VAS).

^e^ Frequency of abdominal pain is scored based on the number of days of pain in the last week.

### 2.2 The expressions of miRNAs in IBS-D patients

#### 2.2.1 Differentially expressed miRNAs

**FIGURE 1:**
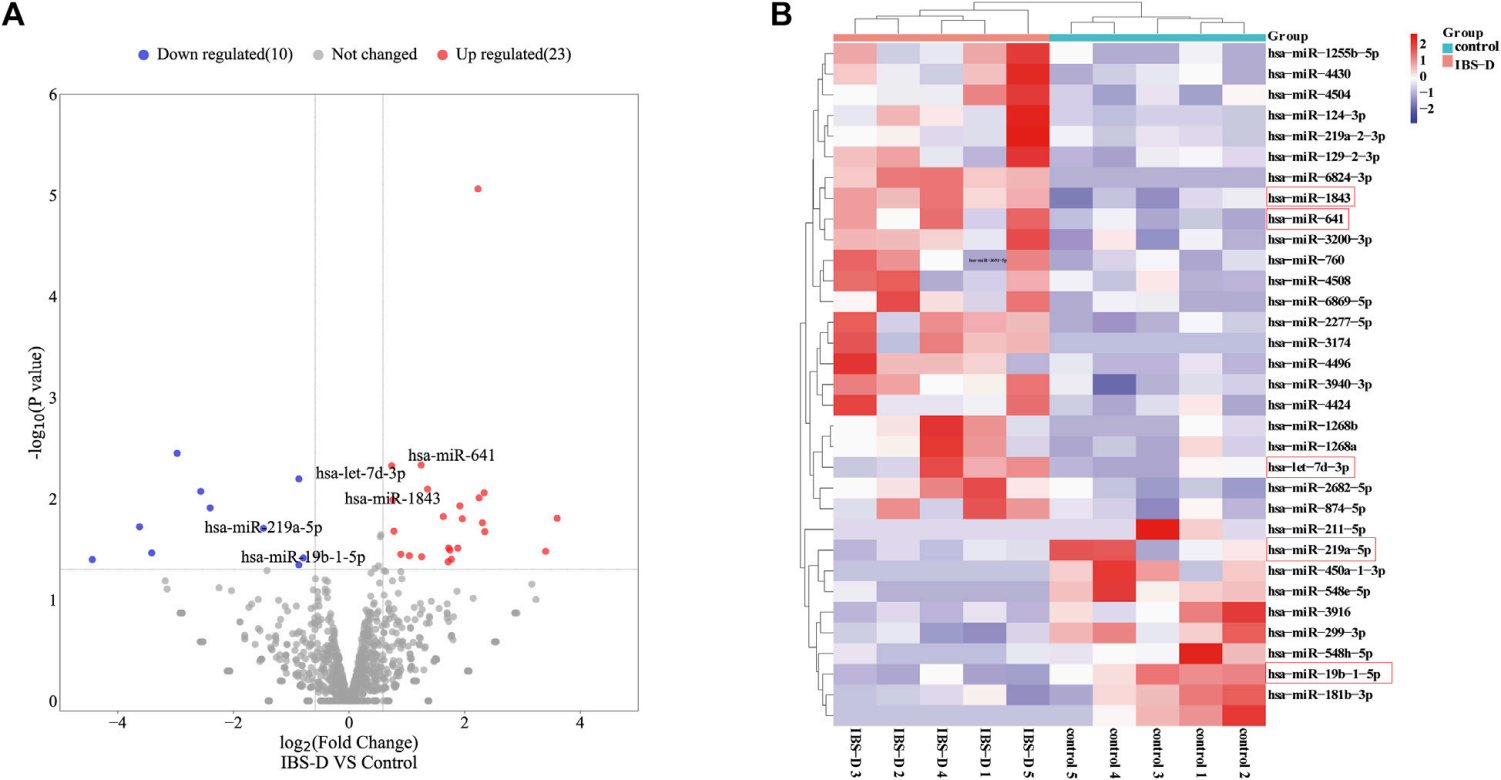
Differentially expressed miRNAs in IBS-D. **(A)** Volcano map of the differentially expressed miRNAs. IBS-D group versus control group. (Red: upregulated miRNAs; Blue: downregulated miRNAs; Grey: non-differentially expressed miRNAs). **(B)** Heat map of the 33 differentially expressed miRNAs with *p* < 0.05 (Red box: miRNAs were found to be expressed in a significantly different manner).

**FIGURE 2:**
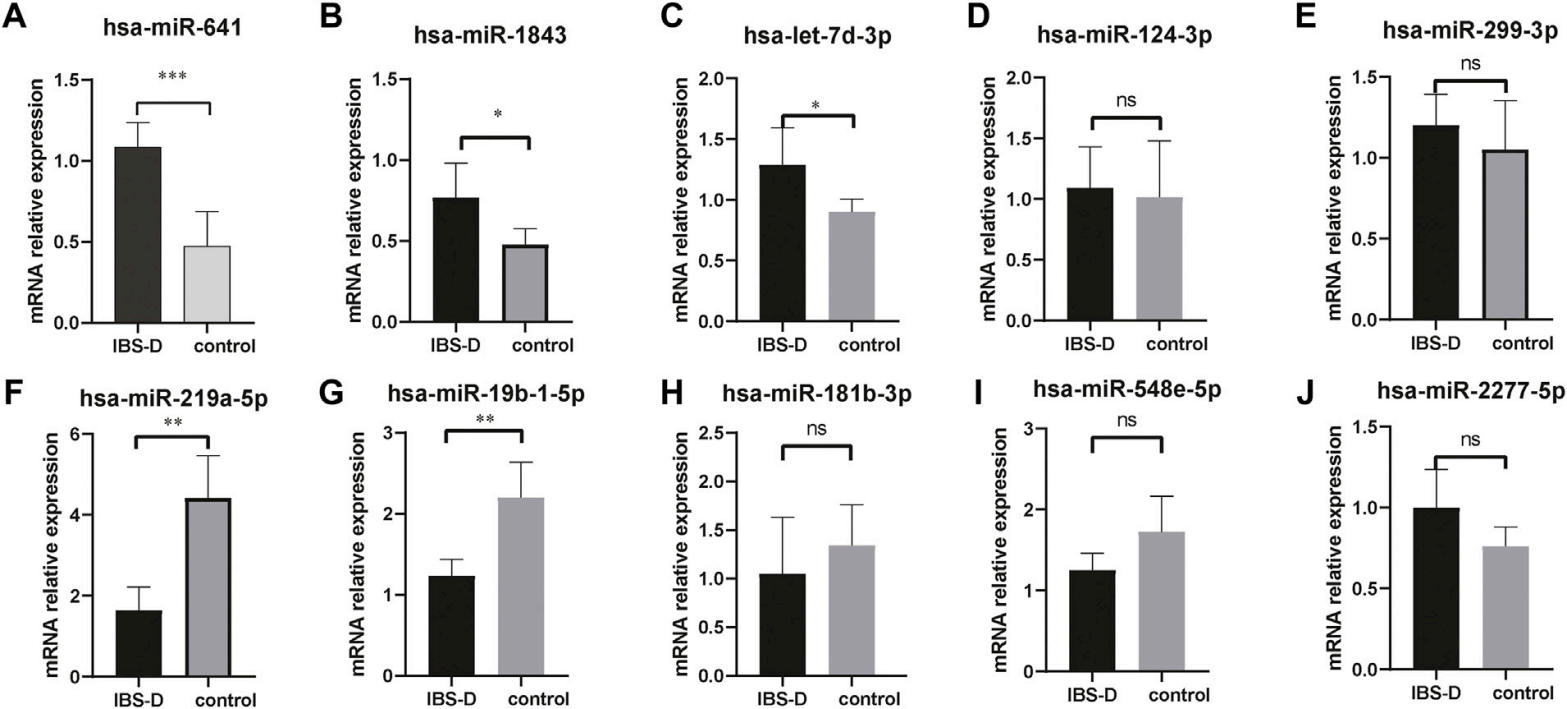
q-PCR verification of differential miRNAs. The mRNA expressions of hsa-miR-641, hsa-miR-1843, and hsa-let-7d-3p were significantly increased in IBS-D patients **(A–C)**. The mRNA expressions of hsa-miR-219a-5p and hsa-miR-19b-1-5p were significantly reduced **(F,G)**. No significant differences for other miRNAs **(D,E,H,I,J)**. * *p <* 0.05; ** *p <* 0.01; *** *p <* 0.001. IBS-D group versus control group.

Small non-coding RNAs with 17-25nt were isolated from tissue samples and the miRNA profile was obtained by high-throughput sequencing. A total of 1883 differentially expressed miRNAs were identified (Supplementary Appendix 1), as shown in Figure 1A. The fold change (FC) > 1.5 and *p*-value > 0.05 were established as the cut-off points. There were 33 differential miRNAs, with 23 upregulated and 10 downregulated, as shown in Figure 1B. The raw sequencing data has been submitted to the GEO database [GEO accession number: GSE212720 (link: https://www.ncbi.nlm.nih.gov/geo/query/acc.cgi?acc=GSE212720)]. By q-PCR experiments of ten differential miRNAs, we found that the expressions of hsa-miR-641 (*p* < 0.001), hsa-miR-1843 (*p* < 0.05), and hsa-let-7d-3p (*p* < 0.05) were significantly increased in the IBS-D group, while the expressions of hsa-miR-219a-5p (*p* < 0.01) and hsa-miR-19b-1-5p (*p* > 0.01) were significantly reduced. Nevertheless, the expressions of other miRNAs were not significantly different. The results were displayed in Figure 2.

#### 2.2.2 The target mRNA prediction and functional enrichment

**FIGURE 3:**
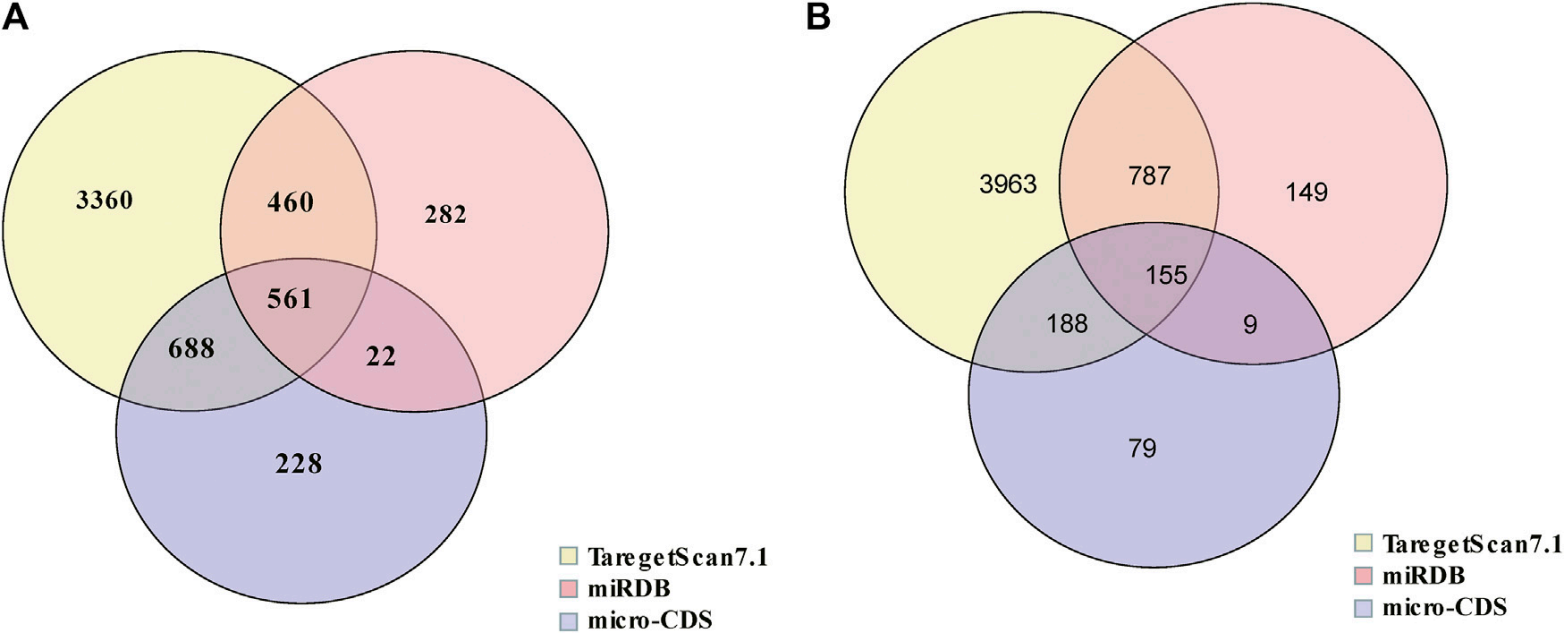
Venn diagram of target mRNAs of differential miRNAs. **(A)** The target mRNA prediction of upregulated miRNAs. **(B)** The target mRNA prediction of downregulated miRNAs.

In order to evaluate the potential function of these miRNAs in IBS-D, target mRNA prediction was performed. The result indicated there were 561 target mRNAs for upregulated miRNAs (Figure 3A) and 155 target mRNAs for downregulated miRNAs (Figure 3B). Detailed data were listed in Supplementary Appendix 2.

**FIGURE 4:**
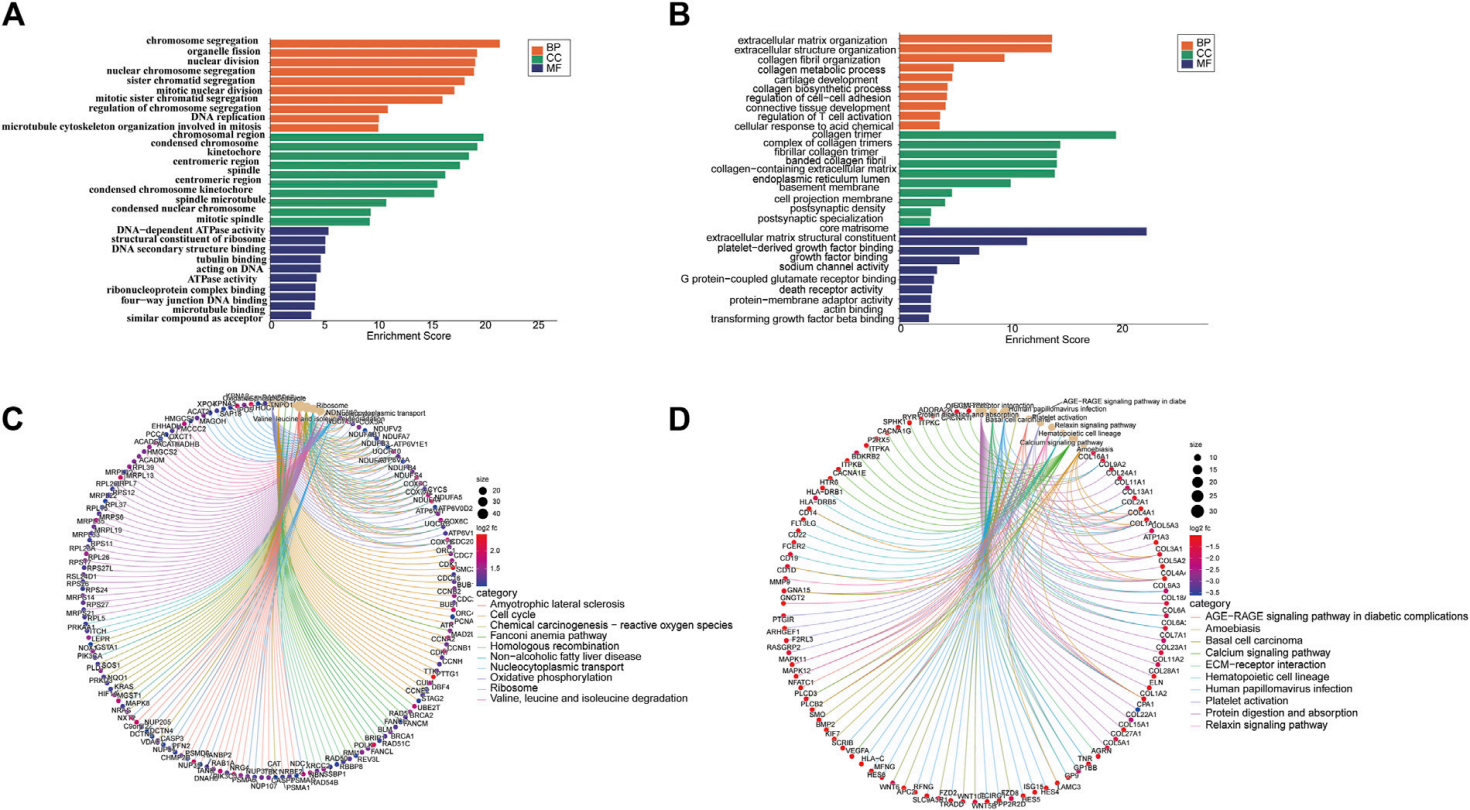
The GO analysis and KEGG pathway enrichment of target mRNAs of differential miRNAs. **(A)** The top five enrichments from Go terms (BP/CC/MF) for the target mRNAs of upregulated miRNAs. **(B)** The top five enrichments from Go terms (BP/CC/MF) for the target mRNAs of downregulated miRNAs. **(C)** The top 10 pathways enriched from KEGG of target mRNAs of upregulated miRNAs. **(D)** The top 10 pathways enriched from KEGG of the target mRNAs of downregulated miRNAs (*p <* 0.05). Coloring indicates the *p*-value with higher values in red and lower values in blue, and the lower *p*-value indicates that the gene is more significantly enriched. (GO, gene ontology; KEGG, kyoto encyclopedia of genes and genomes; BP, biological processes; MF, molecular function; CC, cellular component).

**FIGURE 5:**
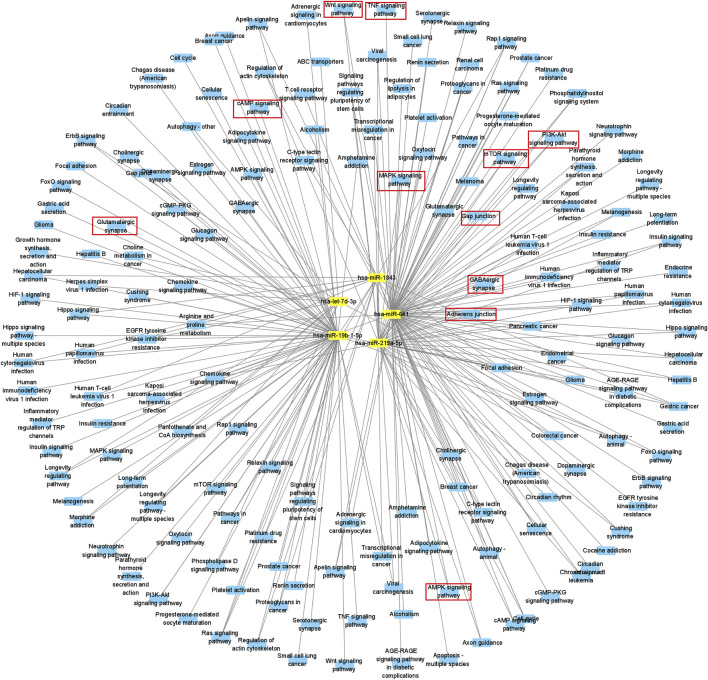
Diagram of a differential miRNA-pathway network. MiRNAs are symbolized by yellow squares, miRNA-related signaling pathways are filled with blue circles, and parts of critical key signaling pathways are marked with red boxes.

From the Gene Ontology (Go) analysis, the biological process (BP) of target mRNAs of the upregulated miRNA was primarily enriched in intracellular signal transduction and protein ubiquitination. Whereas the most notable enrichments of molecular function (MF) and cellular component (CC) were mainly in protein binding and axon, respectively (Figure 4A). Downregulated miRNAs primarily affected the activity of transcription factors and their target mRNAs were abundant in the cytoplasm (Figure 4B). According to the Kyoto Encyclopedia of Genes and Genomes (KEGG) analysis, the target mRNAs of upregulated miRNAs were related to many signaling pathways, including the MAPK, the Wnt signaling pathway and the PI3K-Akt signaling pathway (Figure 4C). While the target mRNAs of downregulated miRNAs comprised signaling pathways such as Melanogenesis, Cushing syndrome, and Growth hormone synthesis, secretion, and action (Figure 4D). By creating miRNA-pathway networks and examining the intersectional signaling pathways enriched by the target mRNAs, we found many important pathways associated with the visceral hypersensitivity of IBS-D, such as the MAPK pathway involved in hsa-miR-641, hsa-miR-19b-1-5p, and hsa-miR-219a-5p, GABAergic synapse and Glutamatergic synapse related with hsa-miR-641 and hsa-miR-19b-1-5p, and Adherens junctions involved in hsa-miR-641 and hsa-miR-219a-5p (Figure 5). These special biological functions and signaling pathways determine the important functions of miRNAs in the epigenetic mechanisms of visceral hypersensitivity.

### 2.3 The expressions of mRNAs in IBS-D patients

#### 2.3.1 Differentially expressed mRNAs

**FIGURE 6:**
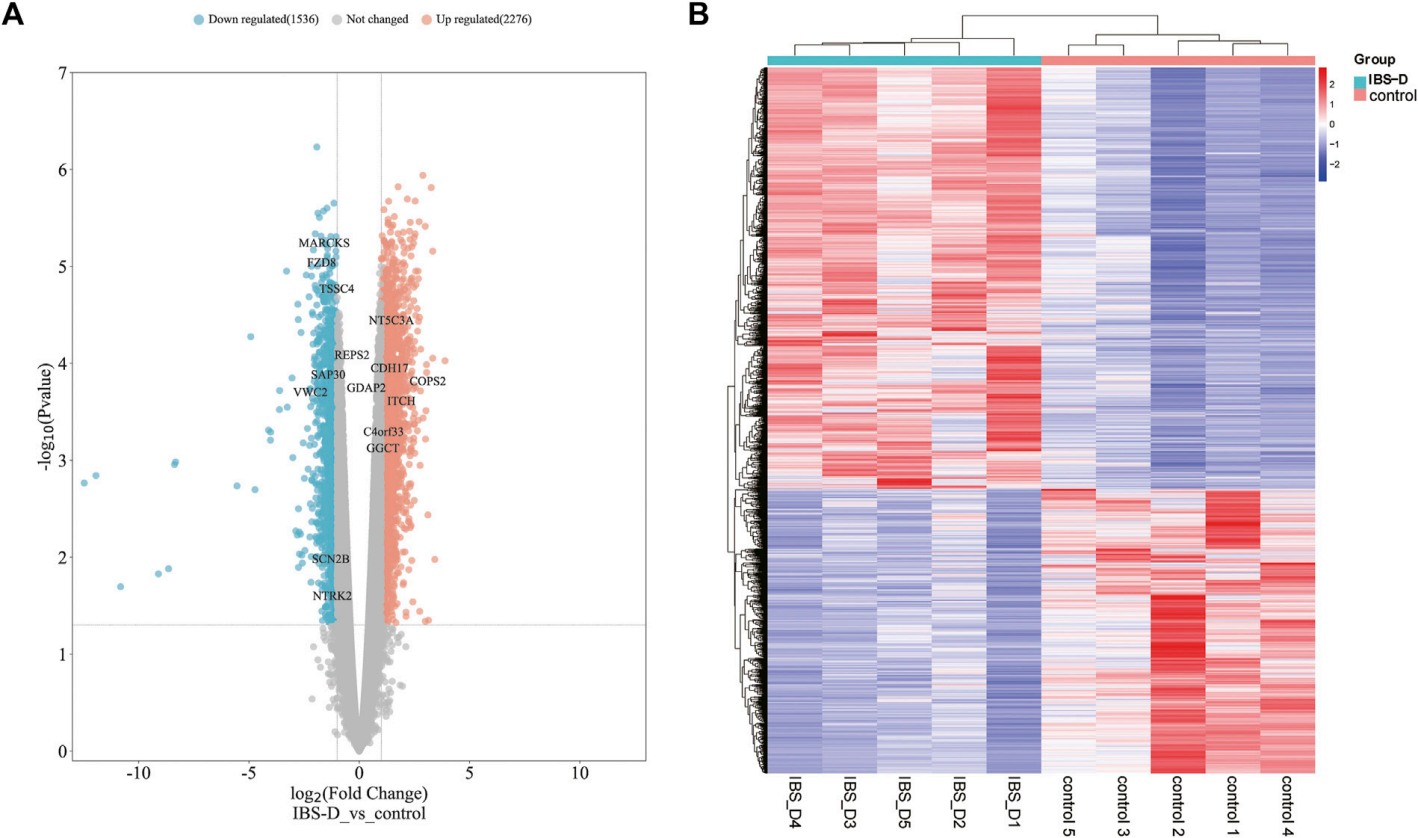
Distribution of differential mRNAs. **(A)** Volcano map of differential mRNAs. Orange indicates the upregulation of genes, cyan indicates the downregulation of genes, and non-differentially expressed genes are shown in grey. **(B)** Heat map of differential mRNAs. IBS-D group versus control group.

A total of 22,679 mRNAs were identified by sequencing. There were 3,812 differentially expressed mRNAs screened with a cut-off point of FC > 1.5 and *p* < 0.05, among which 2,276 were upregulated and 1,536 were downregulated (Supplementary Appendix 3). They were shown by volcano plot and heat map (Figures 6A, B). The raw sequencing data has been submitted to the GEO database [GEO accession number: GSE212719 (link: https://www.ncbi.nlm.nih.gov/geo/query/acc.cgi?acc=GSE212719)].

#### 2.3.2 Functional enrichment

**FIGURE 7:**
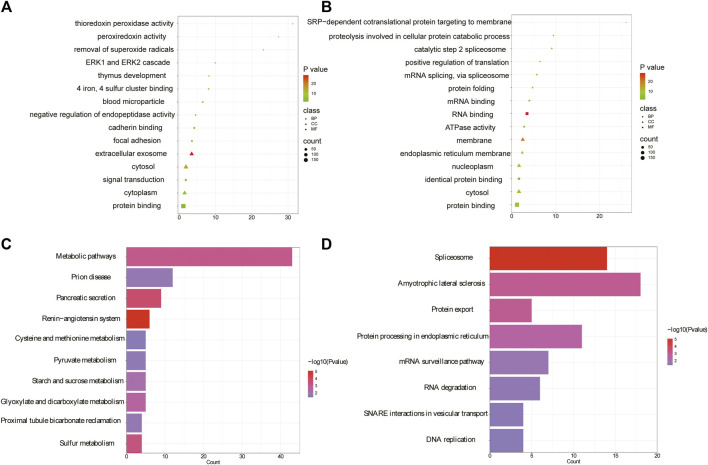
Analysis of GO and KEGG of differential mRNAs. **(A)** The top five enrichments from Go terms (BP/CC/MF) for upregulated mRNAs. **(B)** The top five enrichments from Go terms (BP/CC/MF) for downregulated mRNAs. **(C)** KEGG pathway network diagram for upregulated mRNAs. **(D)** KEGG pathway network diagram of downregulated mRNAs. (GO, gene ontology; KEGG, kyoto encyclopedia of genes and genomes; BP, biological processes; MF, molecular function; CC, cellular component).

The functional analysis of the differential mRNAs revealed that the upregulated mRNAs were significantly enriched in 30 GO terms (Figure 7A), many of which were related to chromosome structure and function. Among them, BP terms were mainly enriched in chromosome segregation, organelle fission and nuclear division. While CC and MF terms were most significantly enriched in chromosome region and ribosome structure, respectively. In addition, for downregulated mRNAs, the BP terms were mainly enriched in the organization of extracellular matrix, regulation of cell adhesion, regulation of T cell activation and collagen metabolic processes. Meanwhile, the most significant MF and CC terms were platelet-derived growth factor binding and collagen trimer, respectively (Figure 7B). KEGG analysis revealed that the upregulated mRNAs were enriched in Oxidative phosphorylation, p53 and mTOR signaling pathway (Figure 7C). The downregulated mRNAs were enriched in Calcium signaling pathway, MAPK signaling pathway and NF-κB signaling pathway (Figure 7D), which have been proved to be associated with visceral hypersensitivity (Mahurkar-Joshi et al., 2021; Zeng et al., 2018).

### 2.4 Bioinformatics function of differential proteins in IBS-D

**FIGURE 8:**
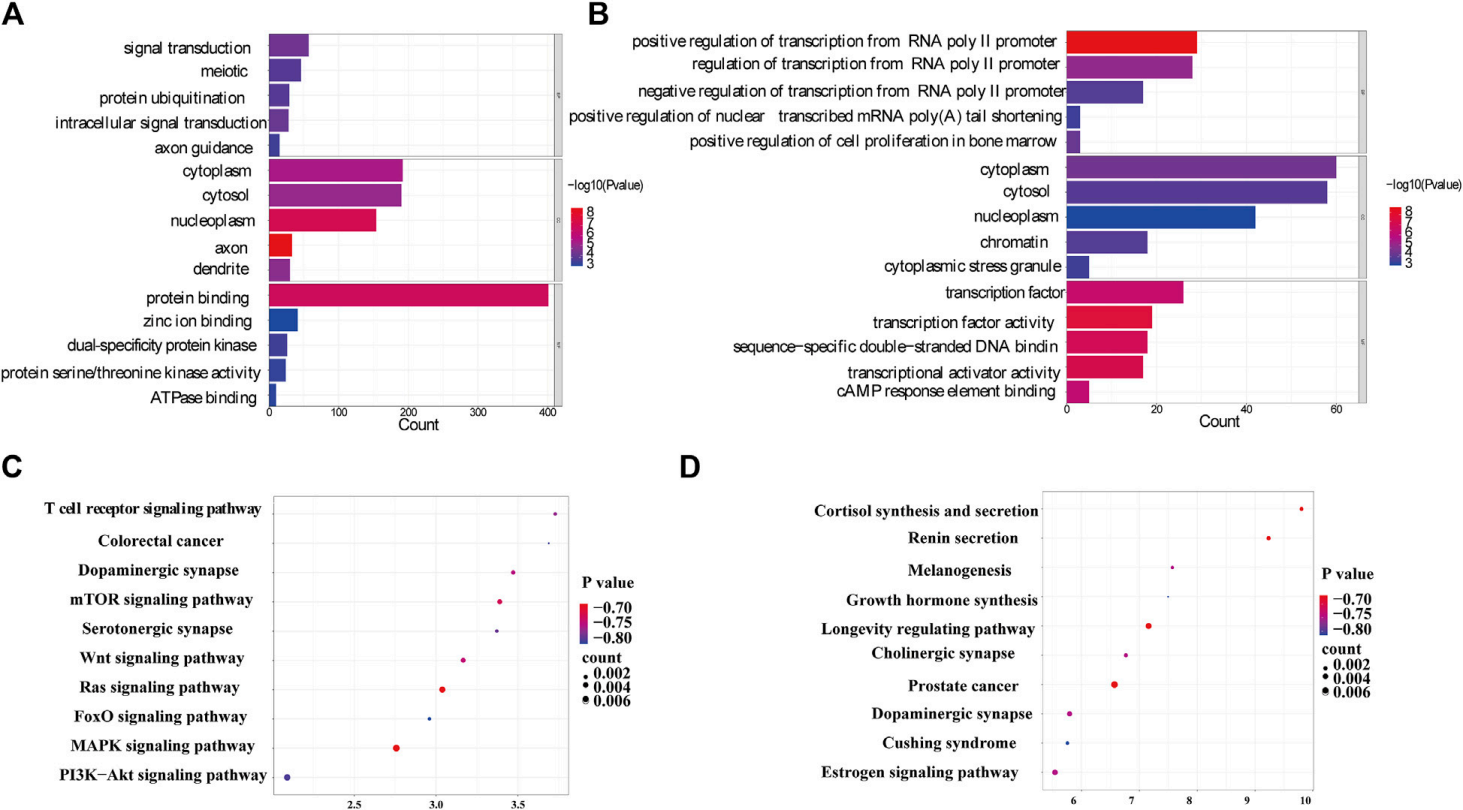
Gene function annotation of differential proteins. **(A)** The top five annotations from GO terms for upregulated proteins. **(B)** The top five annotations from GO terms for downregulated proteins. The color represents significant *p* values, with smaller *p* values resulting in a darker color. **(C)** The top ten pathways from KEGG for upregulated proteins. **(D)** The top ten pathways from KEGG for downregulated proteins. (GO, gene ontology; KEGG, kyoto encyclopedia of genes and genomes; BP, biological processes; MF, molecular function; CC, cellular component).

Previously we found 582 differentially expressed proteins in IBS-D through tandem mass tag (TMT) based proteomics, including 268 upregulated proteins and 314 downregulated proteins (Chai et al., 2021). Here we further investigated the specific functions of above differential proteins by GO and KEGG pathway analysis. The results showed that 81 significant enrichments were identified by GO analysis for upregulated proteins (*p* < 0.05), including BP (23), MF (27), and CC (31). In BP, the upregulated proteins were significantly enriched in signal transduction, negative regulation of endopeptidase activity and regulation of stress-activated pathway. The most significant pathways for MF and CC were calmodulin binding and cellular exocytosis, respectively (Figure 8A). For the downregulated proteins, 127 significant enrichments were obtained, including 45 BP, 27 MF, and 55 CC (as shown in Figure 8B for the top five results). In BP, the downregulated proteins were significantly involved in mRNA splicing complex, protein folding, protein hydrolysis involved in cellular proteolytic processes, etc. MF and CC were mainly involved in RNA binding and membrane separation. KEGG analysis showed that upregulated proteins were enriched in 17 signal pathways including the renin-angiotensin system, pancreatic secretion, etc. (*p* < 0.05). The top ten pathways were shown in Figure 8C. Indeed, downregulated proteins were mainly enriched in eight signal pathways related to genetic information processing, such as spliceosomes and protein exocytosis, as shown in Figure 8D. Intrinsic molecular function and associated signaling pathways of differential proteins were involved in the occurrence of pain perception, such as pressure stress and pancreatic secretion, which were the molecular basis of epigenetic mechanism of visceral hypersensitivity.

### 2.5 Interaction analysis of miRNA-mRNA-protein of IBD-D patients

#### 2.5.1 MiRNA-mRNA interaction network

**FIGURE 9:**
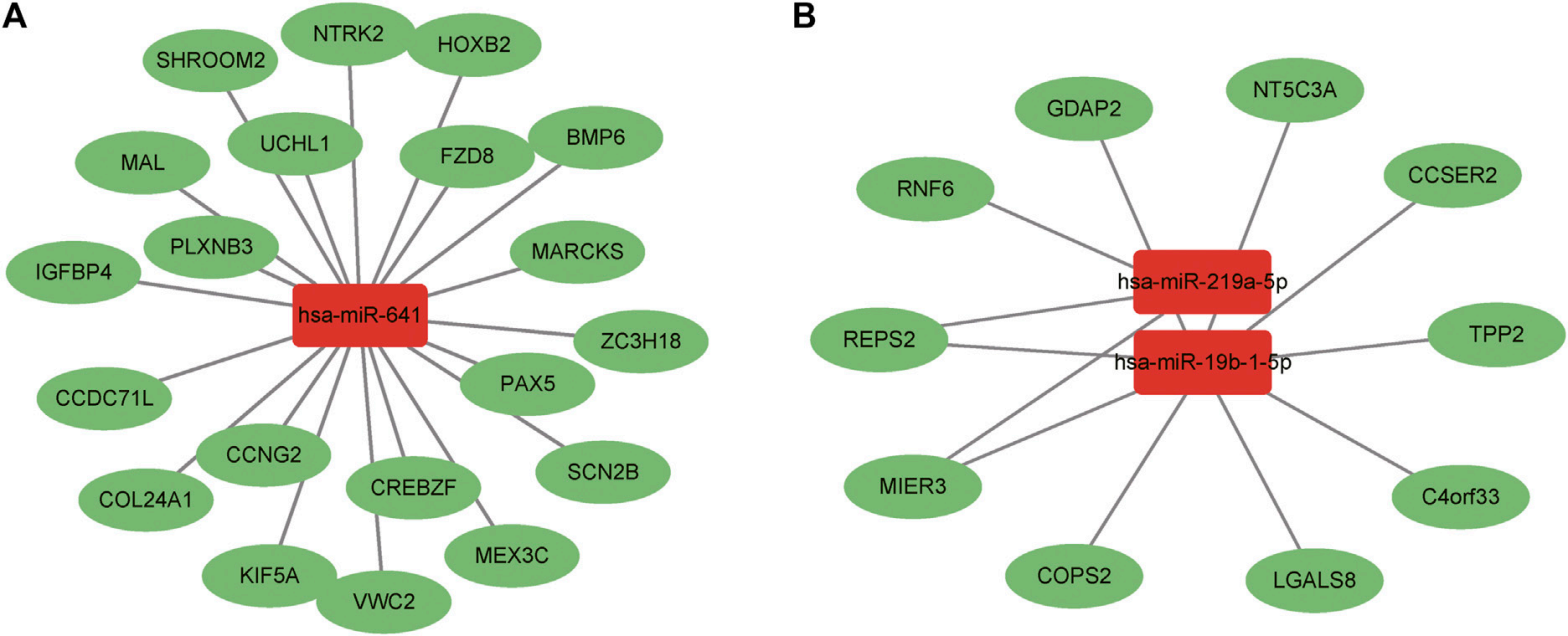
Interaction analysis between differential miRNAs and mRNAs. **(A)** Upregulated miRNAs and downregulated mRNAs. **(B)** Downregulated miRNAs and upregulated mRNAs. Red boxes represent miRNAs and green ovals indicate mRNAs.

Through the combination of the target mRNAs of miRNAs with differential mRNAs, 30 molecules were found to conform to the regulation rule of epigenetics. There were 20 intersecting molecules by analyzing the downregulated mRNAs and the target mRNAs of the upregulated miRNAs, including Sodium Voltage-Gated Channel Beta Subunit 2 (SCN2B), Neurotrophic Receptor Tyrosine Kinase 2 (NTRK2), Von Willebrand Factor C Domain Containing 2 (VWC2), Frizzled Class Receptor 8 (FZD8), and Myristoylated Alanine Rich Protein Kinase C Substrate (MARCKS). While there were 10 intersecting molecules by analyzing the upregulated mRNAs and target mRNAs of the downregulated miRNAs, including Chromosome 4 Open Reading Frame 33 (C4orf33), 5′-Nucleotidase, Cytosolic IIIA (NT5C3A), COP9 Signalosome Subunit 2 (COPS2), Ganglioside Induced Differentiation Associated Protein 2 (GDAP2) and RALBP1 Associated Eps Domain Containing 2 (REPS2). The results were shown in Figure 9.

#### 2.5.2 The interaction analysis of the target mRNAs of miRNA and differential proteins

By analyzing the target mRNAs of miRNA and differential proteins, fourteen intersecting molecules were obtained, including Protein Prenyltransferase Alpha Subunit Repeat Containing 1 (PTAR1), CD44 antigen (CD44), phosphatidylinositol 4-phosphate 3-kinase C2 structure domain-containing subunit α (PIK3C2A), Ras-associated protein Rap-1B (RAP1B), and galactose mutase (GALM). The above protein molecules were inversely related to miRNA expressions, in line with the regulation of miRNAs on targets, as shown in Table 2. These molecules were involved in crucial signaling pathways, such as the MAPK pathway, phosphatidylinositol system, cAMP pathway and Ras pathway (Table 3).

**TABLE 2:** Interaction analysis of target mRNAs of differential miRNAs and proteins.

MiRNAs	Gene symbol	MiRNA type	Protein expression
hsa-miR-641	PIK3C2A	Up	Down
hsa-miR-641	RAP1B	Up	Down
hsa-miR-641	MARCKS	Up	Down
hsa-miR-641	VAMP7	Up	Down
hsa-miR-641	HDLBP	Up	Down
hsa-miR-641	G3BP1	Up	Down
hsa-miR-641	ATP11C	Up	Down
hsa-miR-641	CD44	Up	Down
hsa-miR-641, hsa-let-7d-3p	PTAR1	Up	Down
hsa-miR-641, hsa-let-7d-3p	MTMR6	Up	Down
hsa-miR-19b-1-5p	STEAP4	Down	Up
hsa-miR-219a-5p	PIGR	Down	Up
hsa-miR-19b-1-5p	COPS2	Down	Up
hsa-miR-219a-5p, hsa-miR-19b-1-5p	GALM	Down	Up

**TABLE 3:** Signaling pathways involved in partially interaction protein molecules.

Protein	Type of regulation	Signaling pathway
PIK3C2A	Up	Phosphatidylinositol signaling system
RAP1B	Up	Neurotrophic factor signaling pathway, chemokine signaling pathway, cAMP signaling pathway, Ras signaling pathway, cAMP signaling pathway, MAPK signaling pathway, focal adhesion
MTMR6	Up	Phosphatidylinositol signaling system
GALM	Down	Metabolic pathways

#### 2.5.3 The interaction analysis of differentially expressed mRNAs and proteins

**FIGURE 10:**
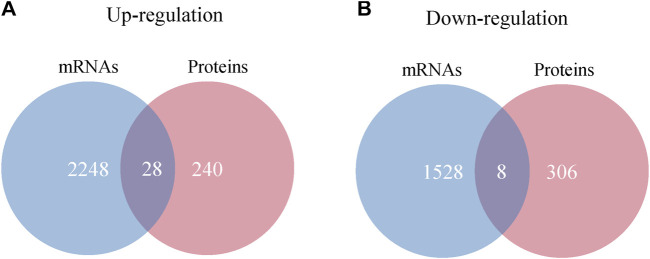
Interaction analysis of differentially expressed proteins and mRNAs. **(A)** Venn diagram of upregulated proteins and mRNAs. **(B)** Venn diagram of downregulated proteins and mRNAs.

By integrating proteomics data and mRNA profile of IBS-D patients, 36 intersecting molecules have been revealed. Among them, there were a total of 28 molecules between upregulated proteins and mRNAs, including Cadherin 17 (CDH17), Carbonic Anhydrase 2 (CA2), Itchy E3 Ubiquitin Protein Ligase (ITCH), and Gamma-Glutamylcyclotransferase (GGCT). There were eight molecules between downregulated proteins and mRNAs, including Sin3A Associated Protein 30 (SAP30), Tumor Suppressing Subtransferable Candidate 4 (TSSC4), CDV3 Homolog (CDV3) and others (Figure 10). All of intersecting molecules were shown in Supplementary Appendix 4.

#### 2.5.4 The interaction analysis of miRNA-mRNA-protein

Two new molecules were identified by interaction analysis of miRNA-mRNA-protein. From the analysis of the target mRNAs of upregulated miRNAs, the downregulated mRNAs and downregulated proteins, intersecting molecule—MARCKS was found to be regulated by hsa-miR-641. While from the analysis of the upregulated mRNAs, upregulated proteins and the target mRNAs of downregulated miRNAs, intersecting molecule—COPS2 was found to be regulated by hsa-miR-19b-1-5p. From both the mRNA and protein expression levels, MARCKS and COPS2 were reversed with miRNAs which were consistent with the regulation rule of epigenetic mechanisms.

## 3 Discussion

IBS-D is a disease without obvious pathological changes and lacks clear molecular markers. At present, the clinical diagnosis of the disease can only be based on the patient’s symptoms, and to exclude organic lesions with the help of colonoscopy and other examinations. These shortcomings greatly increase the difficulty of identifying IBS-D and reduces the adherence of patients to treatment. Therefore, specific molecular markers have always been desired for the early diagnosis and treatment of IBS-D patients.

The study of transcriptional regulatory mechanisms, especially non-coding RNAs, provides an important direction for the discovery of diagnostic markers for a variety of diseases. MiRNAs are abundant non-coding RNA molecules that negatively regulate gene expressions of encoded proteins at the post-transcriptional level by binding with specific target mRNAs. Studies have demonstrated that miRNAs can be used as potential biological markers for the diagnosis of a variety of diseases. In recent years, miRNAs have also been found to function effectively in the regulation of IBS-D gut functions, such as gut microbiota, gut immune response, gastrointestinal barrier function, gut neuronal development and gastrointestinal motility (Gwee et al., 2019; Martinez et al., 2017; Singh et al., 2021; Zhou et al., 2015; Zhou et al., 2016), which are closely related to intestinal visceral sensitivity. Some special miRNAs were associated with a variety of cellular processes, including neuronal plasticity and neurogenesis, injury receptor excitability and visceral pain conditions. The deregulation of some sensory neurons, ion channels and proteins were reported to contribute to the development of central and peripheral sensitization that was considered as the cause of visceral pain (Ji et al., 2003). MiRNAs have the potential to regulate gene and protein expression associated with the induction and chronification of pain (Tan et al., 2013), which form the basis of their involvement in chronic visceral pain condition. MiR-200a may induce visceral nociceptive hypersensitivity through downregulation of cannabinoid receptor 1 (CNR1) and serotonin transporter (SERT), leading to the development of IBS-D in mice (Hou et al., 2018). In IBS patients with increased intestinal permeability, miR-29a expression was increased in blood, small intestine and colon tissues. MiR-29a had a complementary site in the 3′-UTRs of the glutamine ligase gene, which could lead to reduced glutamine synthetase levels, increased intestinal permeability and chronic visceral pain in IBS patients (Zhou et al., 2010). Previously, we found that miRNA-29a could influence the onset of visceral hypersensitivity by targeting HTR7 (Zhu et al., 2019). In addition, our study also certified that inhibition of miRNA-29a regulated intestinal barrier function in IBS-D by upregulating ZO-1 and CLDN1 (Zhu et al., 2020). As well known, increased intestinal permeability is a non-negligible factor for visceral hypersensitivity in IBS-D. Triggered mucosal immune response could cause persistent diarrhea and abdominal pain in patients with IBS (Zhou et al., 2009). One study showed that expression of TRPV1 in the colonic mucosa was enhanced in IBS-D patients and correlated with the severity of abdominal pain (Cheng et al., 2022). At the same time, decreased miR-199 augmented visceral pain in patients with IBS through translational upregulation of TRPV1 by affecting visceral hypersensitivity (Zhou et al., 2016). MiR-16 and miR-125b could affect intestinal barrier function by regulating the expression of tight junction proteins (Martinez et al., 2017). Recent advances found that miR-219a-5p and miR-338-3p could alter barrier function and visceral hypersensitivity in IBS-D patients (Mahurkar-Joshi et al., 2021), further supporting the transcriptional regulation function of miRNAs on visceral hypersensitivity in IBS-D.

From the high-throughput sequencing of intestinal samples of IBS-D patients, 33 differential miRNAs were found, with 23 upregulated and 10 downregulated. Hsa-miR-641, hsa-miR-1843, and hsa-let-7d-3p were proved to be upregulated by q-PCR experiment. PI3K/AKT was one important target pathway of hsa-miR-641 and linked to colonic hypersensitivity (Hinske et al., 2017). Hsa-miR-641 inhibited the PI3K/AKT pathway by downregulating kinases required for AKT2 activation, resulting in neurological dysfunction (Cong and Kong, 2020). PI3K/Akt modulated spinal cord plasticity in visceral pain models independently (Mahurkar-Joshi et al., 2021) and the PI3K cascade reaction could affect intercellular integrity and tight junction (TJ) proteins. After PI3K was inhibited, TJ permeability increased, leading to intestinal dysfunction (Kay et al., 2013). What’s more, hsa-miR-641 was discovered to be increased in inflammatory diseases (Diaz-Prado et al., 2012). Hsa-miR-1843 was shown to be considerably elevated in immunological modulation of bone marrow mesenchymal stem cells (Park et al., 2018). However, there are few studies on epigenetic regulatory mechanisms of hsa-miR-1843 at present and it remains to be discovered whether it is correlated to visceral sensitivity. The expression of hsa-let-7 in intestinal epithelial cells (IEC) was increased, which could inhibit cell proliferation and damage the intestinal epithelial barrier. Our study result was consistent with the increased hsa-let-7d-3p expression level in IBS-D patients in this study. Additionally, hsa-let-7d-3p is a let-7 family subtype that can control a large number of immune genes. Its targets, such as TLR4 and AGO1, played significant roles in several immune responses (Wu et al., 2021). Hsa-let-7 was highly expressed in patients with fibromyalgia and closely related to intraepidermal nerve fiber density and chronic pain (Leinders et al., 2016). In addition, hsa-let-7 could couple with TLR7/TRPA1 channels in sensory neurons to activate nociceptive neurons and cause visceral pain (Ouyang et al., 2022; Park et al., 2014). Nevertheless, more studies are needed to determine whether the immunomodulatory action of hsa-let-7d-3p is related to visceral discomfort in IBS-D. In contrast to the results described earlier, hsa-miR-19b-1-5p and hsa-miR-219a-5p were significantly downregulated by q-PCR experiment. Hsa-miR-19b-1-5p has been shown to be associated with the JAK/STAT signaling pathway as well as neuronal function (Niu et al., 2022), which could restore intestinal homeostasis in mice with intestinal epithelial cell deficiency (Gonneaud et al., 2021), but its function in IBS-D visceral hypersensitivity is unclear. Hsa-miR-219a was significantly reduced in the mouse model of chronic mild stress (Buran et al., 2017) and enabled to activate central pain sensitization by targeting calmodulin-dependent protein kinase II γ (CaMKIIγ), which could regulate BNDF expression and is involved in neuronal plasticity (Hu et al., 2016; Wang et al., 2017). Martinez et al. (2017) found that hsa-miR-125b-5p and hsa-miR-16 could regulate the expression of TJ genes CGN and CLDN2, respectively, and negatively correlated with defecation pattern and the frequency of abdominal pain. Recently, one study reported that hsa-miR-219a-5p was able to change intestinal barrier function and visceral hypersensitivity through neuron and MAPK signaling, so it was expected to be a potential therapeutic target for IBS (Mahurkar-Joshi et al., 2021), strongly supporting the thesis of our study.

Visceral pain of IBS-D is related to the changes of gut microbiota. Gut microbiota could regulate intestinal mucosal immunity and maintain intestinal homeostasis in the body (Liu et al., 2021). The imbalance of gut microbiota could cause intestinal dysfunction, which would lead to visceral hypersensitivity and intestinal motility disorders (Wei L et al., 2021). It has been proved that gut microbiota could significantly influence visceral pain and visceral nociception (Pusceddu and Gareau, 2018). At the same time, gut microbiota also could impact on gut function by regulating neurotransmitters such as 5-HT, whose receptor antagonism has been proved to reduce visceral pain, slow colonic transport and enhance absorption in the small intestine (Camilleri, 2002; Kwon et al., 2019). Interactions between miRNAs and gut microbiota played a key role in homeostasis or dysbiosis of the gastrointestinal environment (Viennois et al., 2019). Hsa-let-7 enhanced Paneth cell differentiation in response to gut microbiota status (Park et al., 2017). A study confirmed that the let-7/TLR4 signaling pathway could ameliorate intestinal inflammation induced by adherent-invasive E.coli (Guo et al., 2018). MiRNA-gut microbiota interaction had been reported in IBD (Casado-Bedmar and Viennois, 2022).

In order to further explore the specific transcriptional regulation mechanisms of these differential miRNAs in intestinal function, we integrated the KEGG pathways involved in the miRNAs, mRNAs and proteins, and then constructed miRNA-pathway relationship network. Some important pathways such as MAPK signaling pathway, GABAergic synapse, Glutamatergic synapse, and Adherens junctions, were found to be closely related to intestinal functions.

The MAPK signaling pathway is an intersecting signaling pathway for the target mRNAs of hsa-miR-641, hsa-miR-19b-1-5p, and hsa-miR-219a-5p. It has three main branching routes—the P38, ERK, and JNK, which could be activated by pro-inflammatory factors and played a key role in the development of inflammation (Yang et al., 2013). Studies have demonstrated that the MAPK signaling pathway could regulate immune-related molecules in a rat model of IBS (Li et al., 2015), and played an analgesic role in a somatic pain model (Zhao et al., 2020). Our study also demonstrated that differential miRNAs were enriched in more genes in the MAPK signaling pathway, which functioned effectively in IBS-D (Mahurkar-Joshi et al., 2021).

GABAergic synapse is involved in multiple pathogenesis of central nervous system diseases and is a potential therapeutic target for disease. *γ*-Aminobutyric acid (GABA), an important component of GABAergic synapse, could modulate visceral pain perception by peripheral afferent neurons (Pokusaeva et al., 2017), while the target mRNAs of hsa-miR-641 and hsa-miR-19b-1-5p were both enriched for GABAergic synapse, involved in pain signaling and inflammatory pathways, and played a crucial role in the pathogenesis in IBS (Fourie et al., 2014). We speculated that these differential miRNAs may regulate visceral hypersensitivity in IBS-D through the GABAergic synapse pathway.

Glutamatergic synapse, as a major excitatory synapse in the brain, is an intersecting signaling pathway for the target mRNAs of hsa-miR-641 and hsa-miR-19b-1-5p. Visceral hypersensitivity in IBS patients was proved to be associated with the regulation of glutamatergic synaptic signaling along the gut-brain axis, which might be responsible for afferent neuronal adaptation and pain processing (Banfi et al., 2021). In this study, we identified that glutamatergic synaptic pathway was associated with different miRNAs, which may be one of the epigenetic mechanisms underlying the visceral hypersensitivity of IBS-D.

Adherens junction, as an intersecting pathway of hsa-miR-641 and hsa-miR-219a-5p, is essential for maintaining barrier function and integrity of epithelium. It has been reported that the expressions of Adherens junction protein E-cadherin, TJ proteins ZO-1, and claudin-1 were all associated with IBS symptoms (Wilcz-Villega et al., 2014). In addition, decreased E-cadherin was related with high abdominal pain score in IBS-D patients and hypersensitivity in rats (Zhang M et al., 2020). However, epigenetic regulation mechanisms of miRNAs on Adherens junction still need to be investigated.

By analyzing the target mRNAs of miRNAs and differential mRNAs, 30 intersecting mRNAs were found and they were involved in a number of neurological biological functions. For example, NTRK2, a member of the neurotrophic tyrosine kinase receptor (NTRK) family, interacted with the downstream effector SOLL1, and then regulated BDNF signaling which has been shown to be associated with visceral hypersensitivity in IBS-D (*Y* Zhang et al., 2019). Then again, SCN2B is a protein-coding gene that encodes a voltage-gated Na+ channel β2 subunit, and it could regulate mRNA and protein expressions of the tetrodotoxin-sensitive α subunit in injury-receptive dorsal root ganglion neurons, ultimately leading to changes in pain sensitivity (Lopez-Santiago et al., 2006). Taken together, we judged that these molecules are likely to be involved in the mechanism of visceral hypersensitivity in IBS-D.

We found 14 intersecting molecules such as RAP1B and CD44 by analyzing differential miRNAs and proteins. RAP1B, as a targeted regulatory molecule of hsa-miR-641, participated in a variety of signaling pathways. RAP1B was a GTP-binding protein with intrinsic GTPase activity. Additionally, RAP1B knockdown could increase endothelial permeability (Ramos et al., 2018) and functioned on establishing basal-endothelial barrier (Pannekoek et al., 2011). RAP1B was also important for early B cell development and homeostasis as well as T-dependent humoral immunity (Chu et al., 2008). However, whether Rap1B is involved in IBS-D visceral hypersensitivity needs to be further explored. CD44 regulated by hsa-miR-641 participated in cell-cell interactions, cell adhesion and migration (Crosby et al., 2009; Vikesaa et al., 2006; Yoshida et al., 2012). This molecule was involved in various cellular functions, including T lymphocyte activation, recirculation and homing, hematopoiesis, inflammation and bacterial infection (Funaro et al., 1994). In conjunction with the results of this study, it can be speculated that CD44 may be associated with immune dysfunction in IBS-D. However, the role of CD44 in the visceral hypersensitivity of IBS-D remains to be verified.

Thirty-six intersecting elements were detected by linking differential expressed mRNAs with proteins. Among them, CDH17, a member of the calmodulin superfamily, can function in the morphological organization of the liver and intestine as involved in intestinal peptide transport (Dantzig et al., 1994). Currently, studies have established that CD17 was more sensitive in the identification of intestinal illnesses (Bian et al., 2017). However, further confirmation is needed whether this molecule is involved in visceral susceptibility to IBS-D. ITCH, as an E3 ubiquitin protein ligase, encoded the proteins that involved multiple cellular processes, such as erythrocyte, lymphocyte differentiation and immune response (Lohr et al., 2010), and was an important regulator of intestinal epithelial homeostasis (Mentrup et al., 2018). But there are few studies on ITCH, so it remains to be verified whether ITCH affect visceral hypersensitivity in IBS-D.

The integrated analysis of miRNA-mRNA-protein revealed two important molecules—MARCKS and COPS2. MARCKS targeted by hsa-miR-641, is a filamentous actin cross-linked protein that inhibits its binding to actin and the plasma membrane. The MARCKS participates in the pathways involved in CNS development and calmodulin binding. One important isoform, MARCKSL1, restricted neuronal activity when phosphorylated by MAPK8 (Bjorkblom et al., 2012). COPS2 regulated by hsa-miR-19b-1-5p is an essential component of the COP9 signalosome complex. It is involved in neuroimmune responses by affecting the activity of NF-κB, JNK and other signaling pathways (Seeger et al., 1998; Uhle et al., 2003). However, its involvement in IBS-D visceral hypersensitivity still needs to be further explored.

## 4 Conclusion

This study integrated all the data of miRNAs, mRNAs and proteins in order to explore the epigenetic mechanism of visceral hypersensitivity in IBS-D from transcription and protein level. The results showed that hsa-miR-641, hsa-miR-1843, hsa-let-7d-3p, hsa-miR-219a-5p, and hsa-miR-19b-1-5p were significantly differentially expressed in the intestinal tissues of IBS-D patients. These five miRNAs could regulate multiple molecules such as MARCKS and COPS2, as well as many important signaling pathways like MAPK and Adherens junction. Our study provided the epigenetic information of IBS-D visceral hypersensitivity from three dimensions. Some of these miRNAs have been intensively elucidated to confirm their role in IBS-D such as hsa-miR-219a-5p (Mahurkar-Joshi et al., 2021), but most of these molecules still have a big gap related with their functions.

However, this study has some limitations. We mainly focused on exploring the relationship between miRNA, mRNA and protein molecules of IBS-D through multi-omics. Although we found some useful miRNAs and their potential effects, due to the small sample size, we failed to comprehensively and strongly confirm their relationship with clinical symptoms. Therefore, multi-center clinical trials with large samples are needed to fully confirm these findings. Besides, the majority of the identified differentially expressed miRNAs, mRNAs and protein molecules have not yet been subjected to in-depth functional study. Whether differential miRNAs can be considered as potential therapeutic targets of IBS-D is needed to explore. Further *in vivo* and *in vitro* experiments are needed to be designed for these key differential miRNAs, mRNA and protein molecules in the development of IBS-D.

## 5 Materials and methods

### 5.1 Clinical data

IBS-D patients and healthy volunteers aged 20–60 were recruited from outpatient and the Physical Examination Center of our hospital. The diagnose of IBS-D was based on Rome IV criteria (disease group) (Gwee et al., 2019). The healthy volunteers served as control group. Prior to the study, all the subjects underwent a complete history and physical examination in order to exclude who had gastrointestinal diseases, chronic diseases, allergic diseases, pregnancy and lactation, as well as who took medicines within the previous 2 weeks. Routine blood analyses, electrocardiogram, and digestive endoscopy were performed to confirm no organic lesions. The study was approved by the Ethics Committee of the First Affiliated Hospital of Zhengzhou University (project approval number: 2019-KY-32). All subjects signed the informed consent form.

The height, weight, age, sex and other basic information of subjects were registered. The bowel conditions were also scored according to the IBS-SSS scale: 1) Defecating frequency is scored based on the average number of bowel movements per day in the last week; 2) Stool consistency is scored based on the Bristol stool pattern score (0 score for type 4, 1 score for type 5, 2 score for type 6, and 3 score for type 7); 3) Abdominal distension is scored on a scale of 0–4 according to severity; 4) Intensity of abdominal pain score according to visual analog scale (VAS); 5) Frequency of abdominal pain is scored based on the number of days of pain in the last week.

### 5.2 Collection of samples

Five IBS-D patients and five healthy volunteers were screened for multiple omics experiment. Tissue samples were taken by the gastroenterologist from the junction of the subject’s rectum and sigmoid colon for biopsies, rinsed in PBS and then quickly stored in liquid nitrogen and transported to the −80°C refrigerator. Repeated freeze-thaw of samples was avoided.

### 5.3 High-throughput sequencing of miRNAs and mRNAs

To get the miRNA/mRNA profiles of IBS-D, we isolated total RNA from tissue samples for high-throughput sequencing. MiRNA sequencing—total RNA samples were first pretreated to remove some RNA modifications that interfered with the construction of small RNA-seq libraries for efficient reverse transcription. Pretreated total RNA from each sample was taken for miRNA-seq library preparation: 1) 3′ adapter ligation, 2) 5′ adapter ligation, 3) cDNA synthesis, 4) PCR amplification, 5) extraction of 134–160 bp PCR amplified fragments. Libraries were denatured to single-stranded DNA molecules and sequencing runs were performed on the NextSeq system and 50 cycles on an Illumina Nextseq 500 sequencer using the NextSeq 500/550 V2 kit (#FC-404-2005, Illumina) according to the supplier’s instructions.

mRNA sequencing—2 μg total RNAs were used for stranded RNA sequencing library preparation using Ribo-off rRNA depletion kit (Catalog NO. N409, Vazyme) and KC-Digital TM Stranded mRNA Library Prep Kit for Illumina^®^ (Catalog NO. DR08502, Wuhan Seqhealth Co., Ltd. China) following the manufacturer’s instruction. The kit eliminated duplication bias in PCR and sequencing steps, by using unique molecular identifier (UMI) of eight random bases to label the pre-amplified cDNA molecules. The library products corresponding to 200–500 bp were enriched, quantified and finally sequenced on Nova-seq sequencer (Illumina).

#### 5.3.1 Data collection and analysis

miRNAs—Diluted libraries were loaded onto kits and forwarded to sequencing runs on the Illumina NextSeq 500 system using the NextSeq 500/550 V2 kit (Cat# FC-404-2005, Illumina). Significantly different miRNAs were selected using the R package edge R, with a cut-off of FC > 1.5 and *p <* 0.05. mRNAs—Reads mapped to the exon regions of each gene were counted by feature Counts (Subread-1.5.1; Bioconductor) and then RPKM was calculated. Genes differentially expressed between groups were identified using the edgeR package (version 3.12.1). *p* < 0.05 and FC > 2 were used to judge the statistical significance of gene expression differences.

### 5.4 q-PCR validation

To further validate the expressions of the differential miRNAs screened by high-throughput sequencing, q-PCR assays of 10 randomly selected miRNAs were performed in five pairs of samples (5 IBS-D/5 Control). C-DNA synthesis was first performed: total RNA was reverse transcribed using Moloney *Murine Leukemia Virus* (MMLV) reverse transcriptase and oligonucleotide-dT primers (Epicentre) at the following conditions: 16°C for 30 min; 42°C for 40 min; 85°C for 5 min, followed by immediate cooling on ice to obtain the reverse transcription products. Reaction conditions: 95°C, 10 min; 40 PCR cycles (95°C, 10 s; 60°C, 60 s). After the amplification reaction, a melting curve of the PCR product was established with conditions: 95°C, 10 s; 60°C, 60 s; 95°C, 15 s and slow heating from 60°C to 99°C. All reactions were performed in three replicate wells. Corrections were performed using RNU6-1 as an internal reference and calculated by the 2^−∆∆CT^ method. Primers: The primers for the differential miRNAs were provided by Shanghai Bollinger Biotechnology Co. Ltd. (Shanghai), and the primer sequences were shown in Table 4.

**TABLE 4:** Primer list.

Gene ID	Primer sequences		Annealing temperature	Length of output
RNU6-1	F	GCT​TCG​GCA​GCA​CAT​ATA​CTA​AAA​T	60	89
	R	CGC​TTC​ACG​AAT​TTG​CGT​GTC​AT		
hsa-miR-124-3p	F	GGGTAAGGCACGCGGT	60	61
	R	GTGCGTGTCGTGGAGTCG		
hsa-let-7d-3p	F	GGG​CTA​TAC​GAC​CTG​CTG​C	60	63
	R	GTGCGTGTCGTGGAGTCG		
hsa-miR-299-3p	F	GGG​TAT​GTG​GGA​TGG​TAA​A	60	64
	R	CAGTGCGTGTCGTGGAGT		
hsa-miR-181b-3p	F	GGG​GGC​TCA​CTG​AAC​AAT​G	60	64
	R	GTGCGTGTCGTGGAGTCG		
hsa-miR-19b-1-5p	F	GGC​GAG​TTT​TGC​AGG​TTT​G	60	65
	R	GTGCGTGTCGTGGAGTCG		
hsa-miR-641	F	GGG​GGA​AAG​ACA​TAG​GAT​AGA​GT	60	67
	R	GTGCGTGTCGTGGAGTCG		
hsa-miR-1843	F	GGG​GGT​TAT​GGA​GGT​CTC​TG	60	64
	R	GTGCGTGTCGTGGAGTCG		
hsa-miR-2277-5p	F	AAAGCGGGCTGAGCG	60	62
	R	GTGCGTGTCGTGGAGTCG		
hsa-miR-548e-5p	F	GCAAAAGCAATCGCGGT	60	61
	R	GTGCGTGTCGTGGAGTCG		
hsa-miR-219a-5p	F	GGG​GGG​TGA​TTG​TCC​AAA​C	60	65
	R	GTGCGTGTCGTGGAGTCG		

### 5.5 Target mRNA prediction of differential miRNAs and enrichment analysis

Target mRNA prediction was performed for differential miRNAs by miRDB, Micro T-CDS, and TargetScan databases. The sequences of the differential miRNAs were entered into the above tools and the optimal prediction results were analyzed. The target mRNAs were further obtained by Fisher’s test and correction.

### 5.6 Bioinformatics analysis

In order to understand the biological functions and signaling pathways in IBS-D of target mRNAs of differential miRNA, mRNAs and protein molecules, GO function and KEGG pathway analyses were performed by the DAVID (http://david.ncifcrf.gov/) and cluster Profiler R packages, respectively. Then, the miRNA-pathway network was analyzed through Cytoscape software version 2.8.3.

### 5.7 Interaction analysis

According to the general rule that miRNAs reverse target mRNAs, the target mRNAs of upregulated miRNAs in IBS-D were correlated with the downregulated mRNA data and proteomic data, while the target mRNAs of downregulated miRNAs were correlated with the upregulated mRNA data and proteomic data, and then the intersection sets were obtained. Based on this, thorough investigation was carried out in order to test distinct molecules regulated by different miRNAs.

### 5.8 Data analysis

Statistical analysis was performed by the software SPSS version 22.0 and GraphPad Prism version 6. Data were presented as mean ± standard deviation. The independent samples *t*-test was applied to analyze sample characteristics of two groups and *p* < 0.05 was considered significant.

## Data Availability

The original contributions presented in the study are publicly available. This data can be found here: https://www.ncbi.nlm.nih.gov/geo/. Accession numbers: GSE212719 and GSE212720.

## Glossary

BP: biological processes
CDH17: cadherin 17
CC: cellular component
CD44: CD44 antigen
C4orf33: chromosome 4 open reading frame 33
COPS2: COP9 signalosome subunit 2
FC: fold change
FZD8: frizzled class receptor 8
GABA: *γ*-Aminobutyric acid
GALM: galactose mutase
GDAP2: ganglioside induced differentiation associated protein 2
GGCT: gamma-glutamylcyclotransferase
GO: gene ontology
IBS: irritable bowel syndrome
IBS-D: irritable bowel syndrome with diarrhea
ITCH: itchy E3 ubiquitin protein ligase
KEGG: kyoto encyclopedia of genes and genomes
MARCKS: myristoylated alanine rich protein kinase C substrate
MF: molecular function
miRNAs: microRNAs
MMLV: Moloney *Murine leukemia virus*
ncRNAs: non-coding RNAs
NTRK2: neurotrophic receptor tyrosine kinase 2
NT5C3A: 5′-nucleotidase, cytosolic IIIA
NTRK: neurotrophic tyrosine kinase receptor
PIK3C2A: phosphatidylinositol 4-phosphate 3-kinase C2 structure domain-containing subunit α
PTAR1: protein prenyltransferase alpha subunit repeat containing 1
RAP1B: Ras-associated protein Rap-1B
REPS2: RALBP1 associated Eps domain containing 2
SAP30: Sin3A associated protein 30
SCN2B: sodium voltage-gated channel beta subunit 2
TMT: tandem mass spectrometry tagging
TSSC4: tumor suppressing subtransferable candidate 4
VWC2: Von Willebrand factor C domain containing 2
