# Supplementary material for: Integrated omics analysis reveals the epigenetic mechanism of visceral hypersensitivity in IBS-D
Source: Front Pharmacol. 2023 Mar 16;14:1062630. doi: 10.3389/fphar.2023.1062630 (PMC10064328; doi:10.3389/fphar.2023.1062630)
Supplement: Supplementary file 1 [file DataSheet1.zip › Appendix 6.pdf]

The First Affiliated Hospital of Zhengzhou University

Ethical review certificate of scientific research project

Ethical review number: 2019-KY-32

Project	Mechanism research of soothing liver and strengthening spleen method on reducing visceral hypersensitivity of IBS-D with liver depression and spleen deficiency via regulating histone acetylation		
Project Funding	National Natural Science Foundation of China		
Project Major	Department of pharmacy	Principal investigator	Yu-na Chai
Method of review	Meeting review	Review time	Apr. 10, 2019
First attendance	13 members : Attendance: 11; Leave: 2; Avoid :0		
Conclusions of the first meeting	Review after necessary amendments		
Second attendance	13 members: Attendance: 12 ; Leave :1; Avoid : 0		
Conclusions of the second meeting	agreed		
List of review documents	Review documents for the first meeting:<br>1. Study Protocol (version number: V1.0, version date: 2019.01.14)<br>2. Informed consent form (version number: V1.0, version date: 2019.01.14)<br>3. Principal investigator's resume<br>4. Recruitment advertisement<br>5. The funding certificate of the project<br>Review documents for the second meeting:<br>1. Informed consent form (version number: V1.1, version date: 2019.03.14)		
Review opinion	This project has passed by the review of the Ethics Committee.		

Note: This review certificate is only used for clinical research purposes of scientific researchers, not for commercial purposes such as product approval for the clinic and product registration with government relevant departments.

The Ethics Committee of Scientific Research and Clinical Trial

The First Affiliated Hospital of Zhengzhou University

Apr. 17, 2019
